# Targeting Apolipoprotein E for Alzheimer’s Disease: An Industry Perspective

**DOI:** 10.3390/ijms20092161

**Published:** 2019-05-01

**Authors:** Georgette L. Suidan, Gayathri Ramaswamy

**Affiliations:** Alzheimer’s Disease and Dementia Research Unit, Biogen Inc., Cambridge, MA 02142, USA; Georgette.Suidan@biogen.com

**Keywords:** apolipoprotein E, Alzheimer’s disease, astrocytes

## Abstract

Apolipoprotein E (apoE), a key lipid transport protein in the brain, is predominantly produced by astrocytes. Astrocytes are the most numerous cell type in the brain and are the main support network for neurons. They play a critical role in the synthesis and delivery of cholesterol in the brain. Humans have three common apoE isoforms, apoE2, apoE3 and apoE4, that show a strong genotype effect on the risk and age of onset for sporadic and late onset forms of Alzheimer’s disease (AD). Carriers of an ε4 allele have an increased risk of developing AD, while those with an ε2 allele are protected. Investigations into the contribution of apoE to the development of AD has yielded conflicting results and there is still much speculation about the role of this protein in disease. Here, we review the opposing hypotheses currently described in the literature and the approaches that have been considered for targeting apoE as a novel therapeutic strategy for AD. Additionally, we provide our perspective on the rationale for targeting apoE and the challenges that arise with respect to “drug-ability” of this target.

## 1. Introduction

Alzheimer’s disease (AD) is the leading cause of dementia in elderly people. Hallmarks of the disease include the deposition of amyloid beta (Aβ) plaques and the presence of neurofibrillary tangles. Thus, strategies that have gained the most traction in the field are predominantly focused on targeting amyloid and tau proteins [1,2]. However, Alois Alzheimer also reported the presence of “adipose inclusions” as one of the pathologies in the AD brain, in addition to amyloid and tau, suggesting that AD brains display signs of aberrant lipid metabolism [3,4]. Emerging data clearly indicate a strong link between AD and lipids. In fact, the brain is the most lipid-rich organ in the body and—despite accounting for about 2.1% of the total body weight—contains about 25% of total body sterols. In the central nervous system (CNS), lipids play a critical role in both structure and function [5,6]. For example, unesterified cholesterol plays a specialized role as the major architectural component of the myelin sheath. In fact, about 70% of total brain cholesterol is present in the myelin sheath. The remaining lipids are present in functionally active pools in neurons and glia. During CNS development, required cholesterol and lipid pools are obtained exclusively from de novo synthesis. Whether there is any influence of plasma lipids on the brain lipid pool is currently unknown. Given such a crucial role of lipids in the CNS, maintaining lipid homeostasis in the brain is critical for maintaining a healthy state. It is therefore not surprising that changes in lipid metabolism in the brain can lead to neurodegenerative diseases. However, the most important association was established in 1994 with the discovery of a profound role for apolipoprotein E (apoE) in AD [7].

Astrocytes are the predominant producers of apoE in the brain [8,9]. In addition to astrocytes, microglia and neurons have also been shown to synthesize apoE [8,10]. ApoE plays an important role in lipid transport to neurons [11,12], synaptogenesis [13], cerebrovascular integrity and cerebral blood flow (CBF) [14,15], hippocampal neurogenesis [16], neuroimmune modulation [17] and amyloid clearance [18,19]. The three common human isoforms, apoE2, apoE3 and apoE4, differ from each other at amino acid positions 112 and 158. ApoE2 has cysteine (Cys) in these two positions, apoE3 has Cys at 112 and arginine (Arg) at 158 and apoE4 has Arg in both positions [20]. These small amino acid differences in the apoE isoforms elicit a profound genotype-dependent effect on apoE function, such as lipid transport and amyloid clearance, among others [20]. Importantly, these three common human apoE isoforms show a strong genotype effect on the risk and age of onset for sporadic and late onset forms of Alzheimer’s disease (LOAD), with apoE4 being the strongest known genetic risk factor for AD.

Despite numerous new genome-wide association studies (GWAS) findings that have been reported, after age, apoE4 is still the strongest known risk factor for AD. While several hypotheses have been proposed, the exact mechanism by which apoE4 contributes to AD is not known. Consequently, there is significant debate on whether apoE should be upregulated or downregulated, with reports showing both protective and pathological roles of apoE4. The goal of this review is to describe the approaches that have been considered to target apoE as a novel therapeutic strategy for AD. 

## 2. Opposing Hypotheses on the Contribution of ApoE4 to AD

### 2.1. Loss of Function

ApoE4 has been suggested to be poorly lipidated relative to apoE2 and apoE3. ApoE protein levels in human cerebrospinal fluid (CSF) are reduced in ε4 carriers compared to ε2 and ε3 by ~30% [21], and the levels of lipid-depleted apoE in ε4 carriers is elevated ~2-fold [22]. Similar results were found in mice expressing human isoforms of apoE. Utilizing mass spectroscopy and enzyme-linked immunosorbent assay (ELISA) methods, it was shown that apoE4-expressing mice have significantly less apoE in their plasma, brain and CSF compared to apoE2 and apoE3 [23]. Proper lipidation of astrocytic apoE to form high-density lipoprotein (HDL)-like particles is essential for its stability and proper function; thus, it is hypothesized that poor lipidation of apoE4 leads to reduced function and downstream pathology [24,25]. Lipidation of apoE in astrocytes is carried out primarily by the adenosine triphosphate (ATP)-binding cassette family member, abca1. Indeed, ATP-binding cassette transporter a1 (abca1) knock-out mice showed reduced levels of brain apoE and lipidated apoE particles in their CSF [25]. These mice have also shown reduced dendritic density in the CA1 region of the hippocampus [26] and increased amyloid burden in the brain when crossed with human amyloid precursor protein (APP)-overexpressing mice [27]. Interestingly, a large scale population study found that a loss-of-function variant of abca1 in humans resulted in reduced plasma apoE and increased risk of AD, cerebrovascular disease and hemorrhagic stroke, suggesting that this mechanistic axis should also be considered for improvement of cerebrovascular health and function [28]. In contrast, abca1 overexpression in mice increases lipidation of apoE and enhances amyloid clearance [29]. Increasing astrocytic abca1 expression and the resulting increase in apoE lipidation has been shown to have beneficial effects on axonal damage in a traumatic brain injury mouse model [30,31]. An elegant study was recently published utilizing astrocytes derived from human inducible pluripotent stem cells (iPSCs) with either ε3/ε3 or ε4/ε4 genotypes, which provided further evidence in human cells that secreted apoE4 is poorly lipidated. Interestingly, astrocytes derived from an ε4/ε4 patient cells did not support neuronal survivalas well as ε3/ε3, further supporting the idea that the reduced lipidation state of apoE4 is tied to neuronal health [32]. Additionally, HDLs have been shown to enhance amyloid clearance through the blood–brain barrier and reduce vascular inflammation, and therefore enhancing apoE lipidation has the potential to be protective to the cerebrovasculature [33,34]. Further supporting data for the apoE4 loss of function hypothesis are the pathological similarities reported between apoE4 and apoE knockout (KO) mice. For example, both strains of mice were shown to have reduced levels of synaptic proteins PSD-95, GKAP and synaptophysin compared to wild-type and apoE3 mice [15]. The inflammatory response of apoE4 mice was similar to apoE KO when challenged with lipopolysaccharide [17]. Further, work in mice that express human apoE isoforms and five familial AD mutations (termed EFAD) concluded that having apoE4 or no apoE leads to increased age-dependent cognitive dysfunction and reduction in synaptic proteins when compared to mice expressing apoE2 or apoE3 [35]. These data collectively support a reduction in function of apoE4 compared to apoE3 or apoE2 and suggest that therapies focused on enhancing the lipid-transporting function of apoE4 would be beneficial for disease treatment. 

### 2.2. Gain of Toxicity

There are several lines of evidence that support the hypothesis that the apoE4 protein plays a toxic role in the disease state. Although, the important role of astrocyte-derived apoE under physiological conditions is not disputed, the potential for apoE (in particular, apoE4) to take on a determinantal role under disease-like conditions has been demonstrated in several animal models. Cerebral ischemia has been shown to lead to expression of apoE in neurons, a phenomenon not detected in control rats [36]. Neuronal-expressed apoE4 is subject to cleavage, resulting in the generation of neurotoxic apoE fragments [37,38]. The identity of the enzyme that cleaves neuronal apoE is unknown, but data support that it is a chymotrypsin-like serine protease [39,40]. Targeting the cleavage of neuronal apoE is a proposed therapeutic approach for AD [41]. Interestingly, it has been determined that astrocyte-derived apoE is not subject to cleavage, suggesting that this therapeutic approach would be specific to neuronal apoE [42]. Further support for this hypothesis was reported in mice expressing the human apoE isoforms crossed with mice which develop tau pathology. ApoE KO mice were protected, whereas apoE4 mice had worsened pathology, suggesting that the apoE4 protein in these mice was exerting a toxic effect [43]. One hypothesis of how apoE4 promotes pathology in AD is through the initial seeding of Aβ [44]. An apoE-inducible expression mouse model bred with a mouse strain expressing the human amyloid precursor protein and presenilin with human mutations (APP/PS) was used to demonstrate that increasing expression of astrocytic human apoE4 enhanced amyloid deposition in mice when expression was turned on prior to initial seeding [45]. These findings were not present in the apoE3 expressing mice, indicating that apoE4 specifically plays a role in seeding of human Aβ [45]. Other reports suggest that apoE4 exerts its toxic effects through diminished receptor recycling due to disruption of endocytic transport. Indeed, it has been shown that apoE4 reduces recycling of critical glutamate receptors (α-amino-3-hydroxy-5-methyl-4-isoxazoleproprionic acid and *N*-methyl-d-aspartate) and apoE receptors which leads to synaptic dysfunction [46,47]. Others have shown that treatment of apoE knock-out neurons with recombinant and lipidated apoE4 leads to an accumulation of insulin receptors in the endosome, which, in turn, reduces mitochondrial respiration and glycolysis [48]. These data supported in vivo work indicating that insulin signaling in the brain was impaired in aged apoE4 target replacement mice [48]. ApoE4 expressed in GABA-ergic neurons was shown to result in memory and cognitive deficits in mice [49]. Additionally, astrocyte-mediated synaptic pruning has been shown to be allele-dependent, with apoE4 astrocytes demonstrating reduced phagocytic capacity, whereas apoE KO astrocytes were similar to apoE3 and wild-type astrocytes, suggesting a gain-of-toxic function of apoE4 [50]. Together, these data support a detrimental role for apoE4 in disease and suggest that therapeutic intervention should focus on removing or altering this toxic isoform.

## 3. Approaches for Targeting ApoE in AD

Although not as mainstream as amyloid and tau approaches, there are approaches for targeting apoE in Alzheimer’s disease being undertaken in academia and industry (Table 1). There are several unique strategies for targeting apoE and clear indecision about the contribution of apoE4 to AD pathology, as mentioned in the previous sections. Below, we highlight some of the latest findings in the field, with the exception of gene therapy and mimetic peptide approaches, which are very intriguing and reviewed nicely elsewhere [51].

### 3.1. Correcting the Structure of ApoE4

Using biophysical and biochemical methods, it has been demonstrated that the presence of Arg at amino acid position 112 in apoE4, instead of Cys 112 as seen in apoE3, results in an interaction between the amino-terminal low density lipoprotein receptor binding domain and the carboxy-terminal lipid binding domain via a salt bridge, leading to a property called domain interaction [58]. Domain interaction has been shown to be responsible for lower levels of secretion of apoE4 from astrocytes relative to apoE3 [59,60]. Additionally, investigators from the Gladstone Institute have shown that when apoE4 is expressed in neurons under stress, domain interaction is responsible for proteolysis of neuronally expressed apoE4 into neurotoxic fragments [6,39,58]. Domain interaction-mediated apoE4 proteolysis was found to result in mitochondrial dysfunction in neurons [41]. Small molecule structure correctors that interfere with this property and convert apoE4 to an apoE3-like molecule have been reported to mitigate the neurotoxic effects [54,55]. Importantly, data generated with structure correctors have exclusively focused on the neuron-expressed pool of apoE, rather than astrocyte-derived. Additional work is necessary to understand the relevance of this strategy for astrocyte-derived apoE4 which does not undergo proteolysis [42]. Whether these structure correctors would increase lipidation of astrocytic apoE4 is also currently unknown. Furthermore, the feasibility of utilizing a small molecule to correct the structure of a secretory protein that undergoes dynamic lipidation processes remains to be seen. Development of small molecule allosteric modulators of apoE4 structure for targeting apoE4-mediated pathology is an interesting strategy and is being pursued commercially by eScape Bio.

### 3.2. Increasing ApoE Lipidation via Abca1

**Figure 1:**
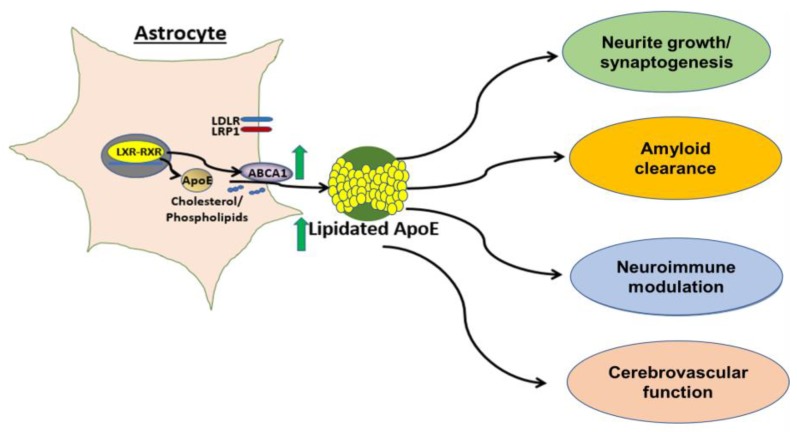
Increasing lipidation of the astrocytic pool of apoE. Astrocytes are the primary producer of apoE in the brain. Common mechanisms of apoE and abca1 expression are driven by nuclear hormone receptor (such as LXR and RXR) activation. Abca1 is critical for lipidation of apoE and increasing expression of abca1 leads to increased lipidation of all apoE isoforms (indicated by green arrows). We hypothesize that increasing apoE lipidation will be beneficial for several AD-relevant endpoints.

Abca1 has been shown to be critical for apoE lipidation [25]. Most known mechanisms regulating expression of abca1 are driven by nuclear hormone receptors, such as liver-X-receptor (LXR)and retinoid-X-receptor (RXR) Indeed, treatment of human astrocytes with small molecule agonists of LXR and RXR results in increased abca1 protein levels in the brain and lipidation of CNS apoE [18,19,61,62]. Hepatic LXRα activation is a key toxicity associated with these nuclear hormone receptor agonists, resulting in fatty liver and increased plasma triglyceride levels in mice [63]. Recently, pan class I histone deacetylase (HDAC) inhibition was shown to increase astrocytic apoE and abca1 in an LXR-independent manner [64]. However, selectivity issues intrinsic to HDAC inhibitors make this class of molecules unattractive for developing therapeutics. Importantly, several groups have demonstrated that lipidation of apoE4 can be achieved pharmacologically, a critical finding when considering this approach to targeting apoE in AD [53]. For example, a direct peptide activator of abca1, CS-6253, has been reported by Artery Therapeutics and researchers at Tel Aviv University to increase lipidation of apoE4 in mouse brain tissue. Further, treatment with this agonist reversed Aβ42 deposition and tau hyperphosphorylation in apoE4-targeted replacement mice [53], suggesting that increasing apoE4 lipidation via abca1 activation could overcome the pathological effects of the apoE4 allele. These data further support work showing that enhancement of apoE4 lipidation can be achieved through nuclear hormone receptor-independent mechanisms. Additionally, a novel chrysanthemic ester was identified that upregulated apoE in fibroblasts deficient in LXRα and LXRβ, indicating an LXR-independent mechanism [65]. Therefore, increasing apoE lipidation is an attractive approach with multi-prong benefits (Figure 1).

### 3.3. Clearing Non-Lipidated ApoE

Recently, an approach was published in which antibodies targeting aggregated, non-lipidated apoE4 were found to reduce amyloid plaques in an APP/PS mouse model crossed with apoE4 knock-in (KI) mice. These studies were the first to show preclinical efficacy with an antibody specific to the non-lipidated form of apoE3 and apoE4 [56]. Currently, it is unknown what the levels and cellular origin of non-lipidated apoE are in the brain and CSF of apoE4 carriers. Interestingly, one study determined that “lipid-depleted” apoE was increased in apoE4 carrier CSF. Whether these antibodies would bind “lipid-depleted” apoE is unknown. The current hypothesis is that this antibody mediates a protective effect through binding of apoE in plaque and inciting a microglial response, leading to clearance. Despite many unanswered questions, this is a novel and interesting approach for targeting apoE.

### 3.4. Reducing Expression of ApoE4

As the gain-of-toxicity hypothesis suggests a deleterious role for apoE4, reducing expression of this protein in the brain is an approach being undertaken. Recently, a study was published that utilized anti-sense oligonucleotide technology to reduce apoE in APP/PS mice crossed with either human apoE3 or apoE4 KI mice. Approximately 50% knockdown of apoE starting at birth resulted in ~50% reduction of plaque area and fibrillar plaque load [57]. Interestingly, knocking down apoE after initiation of Aβ seeding did not alter total Aβ levels and resulted in an increase in average plaque size. These findings bring about interesting questions about the biology and dynamic role of apoE in deposition of Aβ. They also bring to light the challenge of identifying the best age of intervention for an apoE4-lowering therapy. Interestingly, a carrier of a mutation which leads to knockdown of apoE protein was reported in the literature. This was the first apoE null patient to go through extensive cognitive testing and, overall, was largely considered to be normal, suggesting that knockdown of apoE may not be harmful to the brain [66]. However, it was reported that his memory was below what would be considered normal for his age and his language performance was also impaired. Data from apoE KO mice indicate that these mice have age-dependent synaptic loss and cognitive deficits [67,68], however these data could be confounded by the peripheral hyperlipidemia that results from loss of apoE systemically. Interestingly, mice that were deficient in brain apoE specifically did not display deficits in learning and memory, but still showed synaptic loss, similar to total apoE KO [69]. Whether reducing apoE4 expression will have beneficial effects while being safe and tolerable under chronic treatment is a critical question for this approach.

### 3.5. Our Perspective

**Figure 2:**
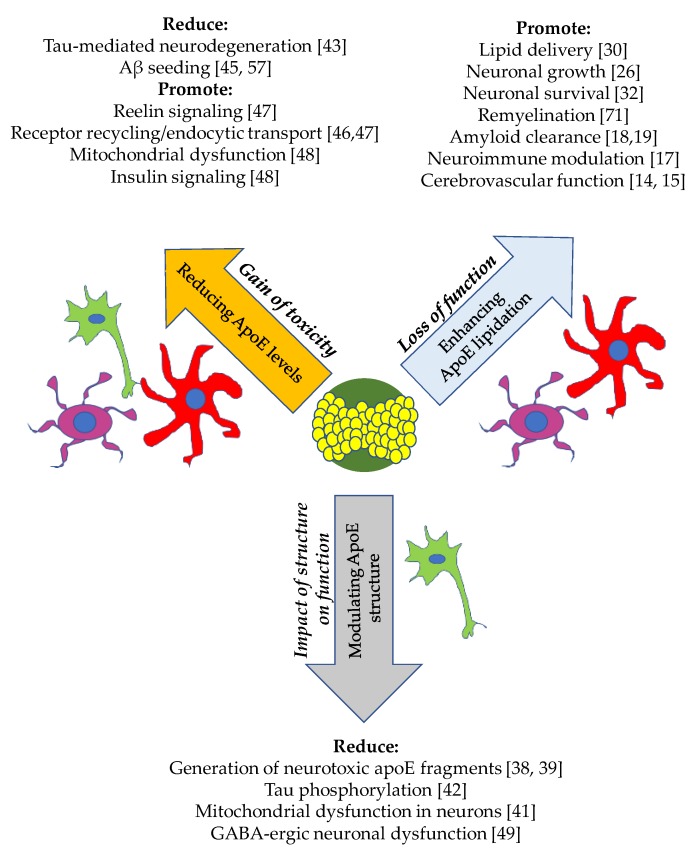
Multiple hypotheses for targeting apoE and their downstream effects. There are many reported findings in the literature which support the hypotheses (italicized) aimed at unveiling the function of apoE in the brain. The approach for targeting apoE in relation to each hypothesis is listed within the arrow. Cell types involved are astrocytes (red), microglia (purple) and neurons (green). A lipidated apoE particle is depicted in the center.

ApoE4 is the strongest known genetic risk factor for Alzheimer’s disease, however, the contributions of apoE to pathology associated with AD are still unclear. Thus, there are many approaches that are being explored in this space that not only differ in modality, but also by mechanism (Figure 2).

Bexarotene, an approved drug for treatment of T-cell lymphoma, was recently tested in a small cohort of AD patients. The goal of the Bexarotene Amyloid Treatment for Alzheimer’s Disease (BEAT AD) study was to test whether the preclinical findings that RXR agonism leads to lowering of soluble and insoluble forms of Aβ in mouse models would be recapitulated in AD patients [52]. Interestingly, treatment with bexarotene resulted in a significant decrease in amyloid positivity by positron emission tomography in apoE4 non-carriers compared to the placebo group, though the groups were exceptionally small. No cognitive benefit was observed, presumed to be due to the short duration of the study. ApoE4 carriers observed no benefit from bexarotene treatment. Modulation of apoE lipidation was not an endpoint in the study, thus is it difficult to conclude from these data whether lipidation of apoE was altered and played a role in decreased amyloid deposition. Interestingly, plasma triglyceride levels negatively correlated with cortical Aβ levels in apoE4 non-carriers. Brain exposure of bexarotene was not reported in this study. Another study was published the same year which evaluated Aβ and apoE metabolism with bexarotene treatment in healthy subjects [70]. This phase Ib study found that brain exposure of orally-dosed bexarotene was in the low nM range while plasma levels were low μM, suggesting low CNS penetration. They reported an increase in CSF apoE but no effect on Aβ metabolism. Lipidation of apoE was not reported due to technical difficulties. Given the hepatic toxicity liability that was demonstrated with LXRα and RXR agonism, identification of an astrocyte-specific mechanism for upregulation of abca1 and apoE lipidation would be advantageous as it would limit associated peripheral toxicity.

Targeting apoE for AD has the potential to influence many aspects of the pathophysiology of the disease. ApoE has been reported to play a role in clearance of Aβ, however, evidence from the literature suggests that the potential beneficial effects of modulation of this protein should not be limited to Aβ clearance. The fundamental role of apoE is to transport lipids. Specifically, apoE is important for delivery of cholesterol to neurons—a cell type which requires cholesterol for maintenance—and for clearance of lipid debris to promote myelin repair. Whether promoting this role of lipidated apoE can be harnessed to improve neuronal function in the disease state has yet to be demonstrated. Interestingly, an LXR agonist has been shown to upregulate abca1 in oligodendrocytes, regulate cholesterol homeostasis and promote remyelination in an animal model [71,72], suggesting that modulation of this mechanistic axis could also be beneficial to demyelination. Furthermore, apoE has been linked to neuroinflammation. ApoE-deficient and apoE4-expressing mice have been shown to have a pro-inflammatory phenotype compared to apoE3 mice after a lipopolysaccharide challenge, demonstrating increased micro- and astro-gliosis and prolonged cytokines production [17]. Additionally, bexarotene treatment in the APP/PS1 mice significantly reduced microgliosis in the hippocampus, suggesting that abca1 and apoE lipidation are important for neuroinflammation [73]. ApoE was recently reported to be a “check point” inhibitor of the classical complement cascade in an isoform-dependent manner where apoE4 results were similar to apoE KO [74]. ApoE binds to, and is a proposed ligand for, the triggering receptor expressed on myeloid cells (TREM2) [75,76], a recently identified candidate risk loci for LOAD [77,78]. This interaction likely modulates AD pathology, though it is currently unclear what the role of apoE is in TREM2 biology; the data are reviewed nicely elsewhere [79].

Although the primary focus of research on brain-derived apoE has been in the AD field, there are more data coming out further exploring the effects of the apoE genotype in cognitively normal people. For instance, there is research on white matter integrity [80] and cerebrovascular reactivity [81], as well as a host of abnormalities reported in apoE knock-in mice, which are reviewed in detail [24]. ApoE biology in the brain is extremely complex with many contradictory findings being reported as to its function and role in the CNS in mice. Therefore, human studies comparing ε4 carriers and non-carriers are more likely to provide information on the roles of apoE in health and disease. Indeed, imaging studies have suggested that ε4 carriers have accelerated brain deformations in the Alzheimer’s disease neuroimaging initiative (ADNI) cohort [82,83]. Interestingly, a recent study conducted in cognitively normal non-carrier, heterozygotes and homozygous ε4 carriers showed a dose effect on hippocampal morphology, with carriers having increased abnormalities in this structure prior to onset of cognitive decline [84]. The results of these studies suggest that having an ε4 allele is detrimental to maintenance of brain structures that are impacted by AD.

Another area of AD biology that apoE most likely plays a role in is cerebrovascular dysfunction [85,86]. The brain is a highly vascularized organ and its high energy demand consumes 20–25% of the body’s oxygen and glucose stores [87]. Blood flow to the brain is required for proper CNS development and is necessary for neuronal viability. Vascular disease was shown to strongly correlate with cognitive dysfunction in AD autopsy samples [88]. Additionally, studies utilizing arterial spin labeling magnetic resonance imaging to assess CBF have reported in the ADNI cohort that deficits occur early in the progression to late onset AD, suggesting that cerebrovascular dysfunction may play a causal role in the development of AD [89]. Interestingly, preclinical data suggest a relationship between the apoE genotype and vascular function, however the mechanisms involved are unclear. Cognitively normal carriers of an apoE4 gene demonstrated impaired cerebrovascular reactivity to hypercapnia assessed by blood-oxygen-level-dependent functional magnetic resonance imaging and transcranial doppler [81,90]. Furthermore, mice expressing human apoE4 have reduced resting CBF [15] and impaired vascular response to neuronal activity (neurovascular coupling) [14]. Blood–brain barrier integrity is also compromised in apoE KO and apoE4 mice [15,91,92]. Lastly, carrying an apoE4 allele leads to increased vascular Aβ deposition [93] and homozygotes were reported to have elevated fibrin(ogen) deposition in Aβ positive vessels [94]. Studies investigating the relationship between apoE and cerebrovascular function are needed to increase our understanding of the role of this protein in vascular dysfunction associated with Alzheimer’s disease.

One clear deficit in the field is the lack of understanding of the relationship between lipidation of apoE and disease endpoints. Increasing lipidation of apoE is an attractive target for drug discovery as it targets the fundamental apoE function, namely lipid transport. However, the association of apoE lipidation status in CSF with markers of AD progression, such as total tau protein, phosphorylated tau, Aβ ratio, and cognitive end points has not been assessed in a large, well-characterized cohort of healthy controls and AD patients. Total apoE protein levels are informative, and are reduced in apoE4 carriers, but do not directly address the question of lipidation status. One reason for this critical gap in the field is the lack of high(er) throughput and quantitative assays to assess protein lipidation, although some advancements have been made for apolipoprotein A–I [95]. Detection of “lipid-depleted” apoE in the CSF of a small cohort of patients [22] has also been published and requires centrifugation over a potassium bromide gradient, which is not ideal for screening a large number of samples. Large scale studies will need to be performed in order to define efficacious dosing for drug discovery programs looking to increase lipidation of apoE.

ApoE biology is highly complex and there are many aspects—such as the cellular origin and lipidation state of apoE—that must be considered when targeting this protein for AD. Additionally, which patient population would most greatly benefit from an apoE-directed therapy and at what stage of the disease it would be most effective are two outstanding questions in the field. In our opinion, addressing these gaps will be critical to the development of effective apoE-directed therapeutics. For example, an apoE lipidation approach may be suitable for all apoE genotypes given the potential of the mechanism to be restorative. However, the correlation of apoE lipidation state with disease pathology needs to be determined. Generation of human data in well-characterized patient cohorts will be critical for addressing this gap. On the other hand, apoE4 reducing approaches may be suitable for apoE4 homozygous patients. Furthermore, work done in apoE KO mice and characterization studies in a null human do not take into account the compensation that may occur in the complete absence of this critical protein in the brain, or the effect it may have on CNS lipid metabolism. Additionally, whether perturbation of peripheral lipid homeostasis in the absence of apoE will lead to vascular dysfunction will need to be determined. Thus, it is currently unclear whether reducing apoE expression in the brain would have a negative outcome with respect to safety. Currently, there are no findings which suggest that increasing lipidation of endogenous apoE in the brain, including apoE4, would carry a safety risk.

In conclusion, apoE is an attractive and rational target for development of disease modifying-therapies for AD. After age, apoE4 is the strongest known risk factor for this disease, and thus targeting apoE could be relevant for those already showing symptoms of the disease. More research is needed in both academia and industry to further uncover the role of this protein in the brain to answer outstanding questions on how it should be targeted.

## Figures and Tables

**Table 1:** Overview of approaches for targeting apolipoprotein E (apoE) for Alzheimer’s disease (AD).

Approach	Rationale	Institution/Company	Risks/Gaps
Bexarotene (RXR agonist; cancer drug-Targetin).	Increase lipidation of apoE.	Indiana University/ADDF [19].	Safety issues due to RXR agonist and impaired brain exposure [52].
Abca1 peptide agonist (derived from carboxy-terminal of apoE).	Increase lipidation of astrocytic apoE4.	Tel Aviv University/Artery Therapeutics [53].	Unclear mechanism by which the peptide agonist activates Abca1, a transmembrane protein.
ApoE4 structure correctors.	Convert neuronal apoE4 to apoE3-like molecule. Mitigate neuronal toxicity caused by apoE4 fragments.	Gladstone Institute/E-Scape bio [54,55].	Based on the premise that neurons express apoE. Effect on astrocytic apoE or apoE lipidation unknown.
ApoE antibody.	Target unlipidated apoE associated with amyloid plaques. Increase amyloid clearance.	Washington University/Denali therapeutics [56].	Based on the premise that unlipidated apoE in associated with plaques. Amount or origin of unlipidated apoE in the brain unknown.
ApoE anti-sense oligonucleotide.	Reduce expression of apoE4 in the CNS.	Washington University/Ionis [57].	Based on the premise that apoE4 is toxic. Safety needs to be assessed as effect of chronic knockdown of apoE4 is not known.
